# Nutritional Considerations in Colorectal Surgery in Diverting Ileostomy Patients: A Review

**DOI:** 10.7759/cureus.48102

**Published:** 2023-11-01

**Authors:** Athanasios Migdanis, Ioannis Migdanis, Georgios D Koukoulis

**Affiliations:** 1 Faculty of Medicine, University of Thessaly, Larissa, GRC; 2 Department of Nutrition and Dietetics, University of Thessaly, Trikala, GRC; 3 Department of General Surgery, General Hospital of Larissa, Larissa, GRC

**Keywords:** dehydration, electrolyte profile, energy balance, nutritional status, ileostomy

## Abstract

Colorectal surgery often results in a temporary or permanent ileostomy construction. The general nutritional status and intake of patients with an ileostomy have not received much attention and scientific evidence is lacking. Nutritional complications associated with ileostomy formation and colonic exclusion include fluid (dehydration) and electrolyte abnormalities (mainly hyponatremia), impaired renal function occurring from plasma volume depletion, and reduced energy absorption due to the role of the large bowel in energy assimilation. People with ileostomies frequently avoid specific foods, due to concerns of possible malfunction or food blockages of their stoma, which may produce a negative effect on their overall dietary intake and nutritional status. The present article reviews the existing literature on nutritional considerations for those with an ileostomy and discusses measures to optimize overall nutritional status of this category of patients.

## Introduction and background

Colorectal surgery is performed for a number of diseases, such as inflammatory bowel disease (commonly known as IBD), colorectal cancer, recurrent diverticulitis, and intestinal obstruction. It may result in a permanent or temporary ileostomy formation as a way to prevent the intensity of complications related to an anastomotic leakage or due to the necessity of removing the entirety of the colon, rectum, and anus (proctocolectomy) [1]. When cancer of the rectum occurs, where low anterior resections are necessary, the majority of patients in surgical practice receive ileostomies, due to the high possibility of anastomotic leakage [2]. Approximately 120,000 ostomies are performed every year in the United States, with an estimated 32.2% (N = 38,640) of them being ileostomies [3]. Perioperative and postoperative complication rates involved with an ileostomy creation vary between 20% and 60% (N = 7728-23,184) [4,5]. Despite the general complications connected with the procedure, the aim of the present article is to discuss the existing literature that concentrates on the main nutritional issues associated with the formation of an ileostomy in patients undergoing colorectal surgery.

Methodology

A literature search was performed using Medline, PubMed, Ovid, Google Scholar, and ScienceDirect databases for all the articles published between January 1980 and December 2022 related to the subject under investigation. The literature search conducted was not restricted to specific languages. The search concentrated on scientific articles that examined nutritional parameters associated with a diverting ileostomy formation and the impact such a procedure can have on the general nutritional status of such patients postoperatively. The following search terms were used: ileostomy, nutritional status, energy balance, dehydration, electrolyte profile, rectosigmoid resection, colorectal surgery, and rectal cancer. Inclusion criteria included articles on ileostomy and the complications connected to it, studies on adult patients, clinical trials including randomized controlled trials, observational studies, systematic reviews and meta-analyses, and narrative reviews. Exclusion criteria included studies conducted on non-adults (<18 years of age), animal studies, studies about other types of stomas such as colostomies or urostomies, and case studies. The articles used were reviewed and assessed in detail by two independent reviewers.

## Review

Dehydration and electrolyte abnormalities

**Figure 1:**
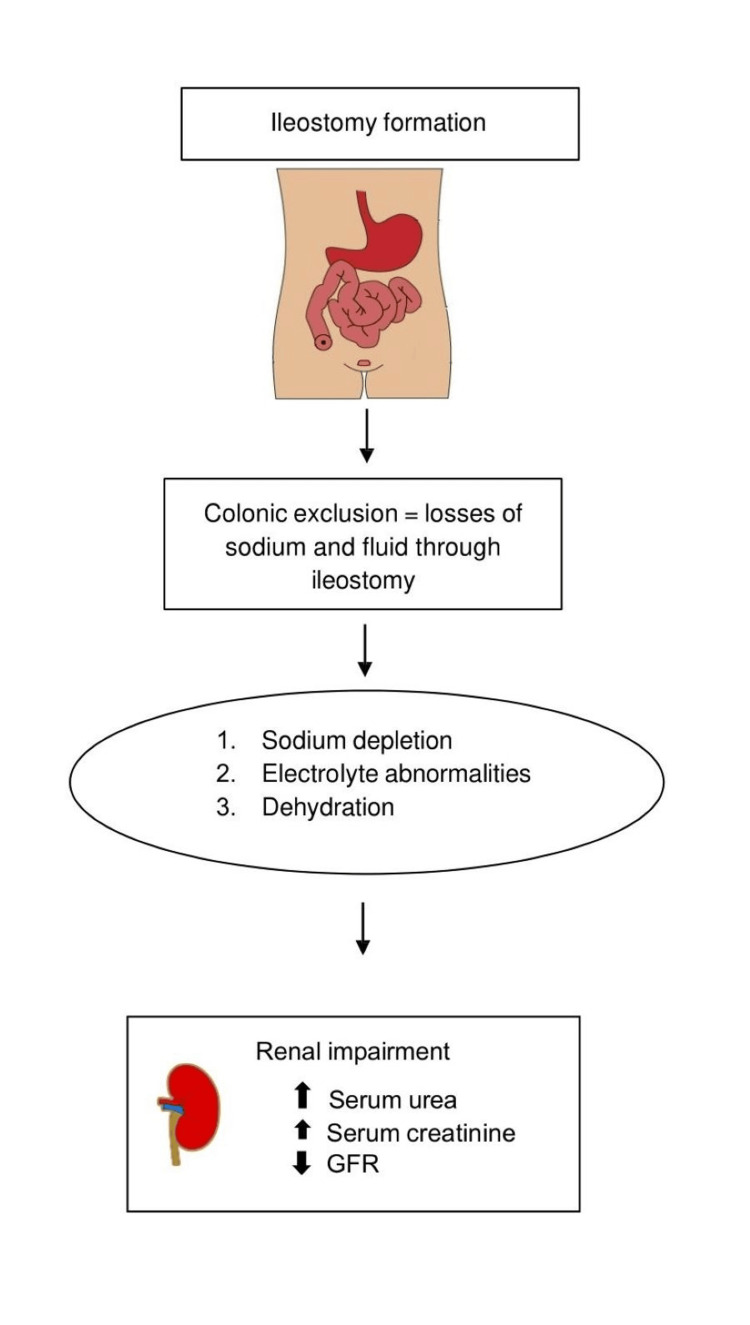
The consequences of a diverting ileostomy formation on fluid and electrolyte equilibrium and renal function. GFR: glomerular filtration rate.

A new ileostomy usually starts to work within 24 hours of formation and initially produces about 1200 mL of watery stool, which reduces in quantity and thickens over the next two to three months. The output may vary from 500 mL per day (mL/d) to 2000 mL/d, according to the volume of food and drink consumed and the volume of gastrointestinal secretions [6]. It is well established that the human colon has a nominal mucosal surface area of about 2000 cm^2^ [7]. In reality, however, the total absorptive area is even greater, because colonic crypt cells are capable of absorption as well as secretion [8]. Its main function is to absorb about 90% of the 1.5-2 L of ileal effluent that passes daily through the ileocecal valve. During this process, water, sodium, and chloride are absorbed, at a greater degree, in the proximal (ascending) segment and, to a lesser extent, in the distal (descending and sigmoid colon/rectum) segment [9]. Due to colonic exclusion, ileostomy patients lose large amounts of sodium and fluid through their stoma effluent (85-180 mmol/L/24 h). This often results in sodium depletion, electrolyte abnormalities, and dehydration [10], as outlined in Figure 1. As far as potassium is concerned, its balance is not often a problem, because the losses from the ileostomy are little [10]. It is important to note that sodium depletion in these cases can increase the production of aldosterone from the adrenal gland, promoting sodium retention and secretion of potassium from the kidney. In patients with a stoma daily output that exceeds 1200-2000 mL, hypomagnesemia can also occur, which can lead to hypokalemia since many of the potassium transport systems can be disrupted and renal potassium excretion can be increased [11]. Thus, rehydration to correct secondary hyperaldosteronism seems to be of prime importance. By restoring sodium and water depletion, potassium levels will improve and usually, potassium supplementation is not required [12].

**Figure 2:**
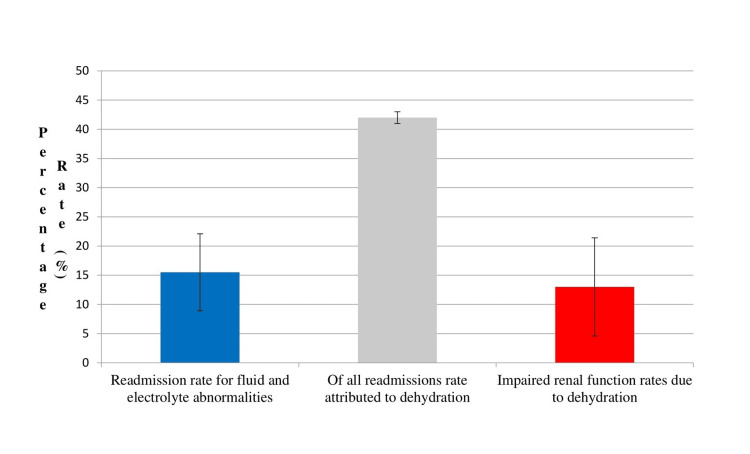
Readmission rates related to dehydration and electrolyte abnormalities in new ileostomates within 30-60 days post-discharge and impaired renal function rates due to dehydration at the time of closure of ileostomy and 30-60 days post-discharge. From [13-17, 26-29].

Among the leading causes of hospital readmission in ileostomy patients are electrolyte and fluid abnormalities. Retrospective studies have suggested a variable occurrence rate [13-17]. In a study by Hayden et al. [13], which included 154 ileostomy patients, the authors observed a readmission rate for fluid and electrolyte abnormalities that reached 20.1% (N = 31). Dehydration accounted for 40.7% of all readmissions and the most common readmission day was day seven post-discharge. The results coincide with another retrospective review by Messaris et al. [14], where 43% of readmissions were attributed to dehydration among the 603 patients who underwent colon and rectal resections with a diverting loop ileostomy over a period of 20 years. The authors also identified the use of diuretics as a sole independent factor associated with dehydration leading to readmission in this group of patients. In a study by Paquette et al. [15] who conducted a retrospective review on patients undergoing ileostomy creation to identify patients readmitted for dehydration or renal failure within 30 days of the surgical procedure, of the 201 patients that participated, 15 patients (8.1%) were admitted for dehydration alone. Multivariate logistic regression indicated that ileal pouch-anal anastomosis (IPAA) surgery was significantly associated with admission for dehydration (OR, 4.95; 95% CI, 1.54-15.83; p = 0.007). Other studies have cited an 11.4% to 15.5% readmission rate for dehydration following ileostomy creation [16,17] (Figure 2 - displays the readmission rates related to dehydration and electrolyte abnormalities in new ileostomates within 30-60 days post-discharge). In a retrospective study [16] from 123 patients who underwent a planned defunctioning loop ileostomy performed over a six-year period synchronously with a colorectal, coloanal, or ileoanal anastomosis, 11.4% (N = 14) were readmitted for dehydration ± acute renal failure. Furthermore, the authors concluded that hypertension and older age can increase the risk of dehydration. In another study from Boston, Massachusetts [17], from 161 patients with new ileostomies for diversion of low anastomosis, primarily in patients with IBD and cancer, the readmission rate for dehydration alone was reported to be 15.5% (N = 25). In both studies, readmissions occurred within 30 days of discharge. 

Managing dehydration

Postoperative information for those with ileostomies should include recognizing the symptoms of dehydration, including thirst, dizziness, lethargy, headaches, and a decrease in skin turgor. Patients with stomal losses of less than 1200 mL/d can usually maintain sodium balance by adding extra salt at the table and when cooking or by consuming salty snacks [18]. Advice frequently offered by medical professionals on increasing overall fluid intake seems improper, as it can dilute sodium levels in the intestinal lumen even more, leading to greater sodium depletion [12,18,19]. It has been suggested that both hypotonic (e.g., tea, coffee, water, and fruit juices low in sugar content) and hypertonic fluids (e.g., fruit juices high in sugar content, Coca-Cola®, and most sip feeds) should be restricted to less than 1 L per day in patients with high output stoma (> 1200 mL/d). To compensate for their fluid requirements, patients should be administered an oral rehydration solution (frequently referred to as ORS) containing at least 90 mmol/L of sodium, throughout the day [6,12,19]. Since sodium and glucose co-transport for absorption in the jejunum, the ORS used should also contain glucose [20]. Previous research concentrating on replacing fluid and electrolyte losses showed that patients with a jejunostomy can achieve a positive sodium balance when administered sodium chloride capsules and oral glucose/electrolyte solutions [21,22]. In administering interventions and analyzing stoma effluents both studies used a crossover approach. In a more recent study patients (n=75) with both high-output ileostomies and jejunostomies (often referred to as HOS-high output stomas) were identified and treated with an HOS protocol that included anti-diarrheal medication, parenteral infusions, oral glucose-saline solution, and oral hypotonic fluid restriction at various stages in a prospective manner [6]. According to the results, the authors stated that if oral intake is restricted and an oral glucose-electrolyte solution is administered, the majority of patients can be treated successfully without parenteral liquids and electrolytes. For patients whose maintenance of hydration remains a challenge, intravenous fluids might be required for a few days. A study that concentrated on preventing rather than treating electrolyte and water imbalances in such patients showed that administering an oral isotonic glucose-sodium drink during the first few weeks after the ileostomy construction can have a prophylactic effect on patients with a newly formed ileostomy, maintaining sodium and renal function markers within normal ranges and preventing readmission for fluid and electrolyte imbalances [23]. Lastly, educating patients about ileostomy function and self-management and encouraging post-discharge daily tracking of intake and output have been proven to decrease dehydration rates [24].

Renal function

Dehydration and disturbances in electrolyte equilibrium in ileostomy patients can result in extracellular fluid volume depletion, as has been well documented in the literature [25]. Reduced blood flow frequently affects acid-base balance and renal function, resulting in patients being readmitted with laboratory evidence of acute renal failure, including elevated blood urea and creatinine levels (Figure 1). In a study by Beck-Kaltenbash et al. [26], the authors prospectively examined the effect of temporary loop ileostomy creation on renal function. Results showed that patients with a diverting stoma had a significantly lower glomerular filtration rate (GFR) at the time of ileostomy closure compared to the time of ileostomy formation. The majority of the patients presented with only moderate decreases in GFR that were not clinically significant; however, 19% of them were found to have clinical renal failure secondary to dehydration. These findings were consistent with the results of a similar study [27], where 21% of participants (n = 308) presented a GFR < 60 mL/min/1.73 m^2^ at the time of closure of the loop ileostomy compared to 7.5% at one week before the ileostomy creation surgery. Again, a significant decrease in mean GFR values using all subjects was noted at the time of closure (89 vs 83 mL/min/1.73 m^2^; P < 0.0001) compared to before the formation of the stoma. Moreover, it was stated that preoperative factors for GFR <60 at closure were age and hypertension.

Other studies focusing on the subject have reported clinically significant declines in renal function or acute renal failure rates ranging from 3% to 17% in such patients [15,28,29] (Figure 2 displays impaired renal function rates due to dehydration at the time of stoma closure and 30-60 days post-discharge). Furthermore, in a case series including five patients with only recently placed ileostomies (few months) and two patients with long-standing ileostomies, readmission for electrolyte and acid-base disorders was observed. All patients presented with increased stoma output (> 1 L/d), extracellular volume depletion, and laboratory evidence of acute renal failure, including increased serum urea and creatinine levels, and an acute decrease in GFR. Recovery of kidney function and resolution of electrolyte disorders occurred when treating the patients with intravenous isotonic saline [25]. Such findings illustrate that patients with ileostomies are vulnerable to developing sudden acute acid-base and electrolyte disorders in association with an increase in ileostomy output. First-line treatment should be vigorous volume repletion with isotonic saline since it is crucial for the restoration of kidney function. After acute management, attempts to decrease output are essential to reduce the likelihood of recurrence. Increased sodium intake without increasing fluid consumption seems to be the answer. Other methods involve administering medication, such as loperamide and codeine phosphate, which can lower intestinal motility and consequently reduce water and sodium output from an ileostomy by approximately 20%-30% [30,31].

Further nutritional considerations

Due to the fact that the major part of macro- and micronutrients are absorbed in the small intestine, ileostomy patients do not often develop any further nutrient deficiencies and should be encouraged postoperatively to follow a regular diet without any restrictions. Foods that are high in fiber and/or harder to digest such as dried fruit, wholegrains, popcorn, nuts, and raw cabbage may get stuck in the intestine causing a blockage or obstruction so it is important to chew food thoroughly [32,33]. If food blockage does occur, it is usually temporary, causing abdominal pain, and the ostomy output will either cease or contain excessive amounts of watery fluid. It has been suggested that if food blockage is suspected but the ileostomy is draining some stool, then oral fluids may be taken but solids should be avoided. However, if there is no effluent being passed, nothing should be taken orally [34]. Generally, the possibility of presenting electrolyte imbalances or nutrient deficiencies is increased in the primary postoperative period (1-3 months), before small intestine absorptive adaption occurs [6,12]. Nutritional requirements differ according to the location of the intestinal resection. Fat and vitamin B12 malabsorption can occur when more than 60-100 cm of terminal ileum has been resected [35,36]. It has also been observed that patients with long-standing ileostomies often develop folic acid and vitamin B12 malabsorption [37].

A few studies have focused on the effect of certain foods on ileostomy function. In one of them [38], the authors analyzed seven-day weighed intakes and 24-hour urine and fecal collections in 36 volunteers with established ileostomies, and compared them to healthy controls. Most patients were in good nutritional health although 47% (n = 17) had an elevated plasma creatinine indicative of renal impairment and 25% (n = 9) had a raised aldosterone concentration. The mean 24-hour ileostomy output for all participants was 760 ± 322 g, with sodium concentration being constant (at approximately 118 mmol/kg). The main determinant of sodium loss was the volume of ileostomy effluent. Of great interest seems to be the fact that participants who were obtaining more of their dietary energy from sugars tended to have reduced effluent and ileal sodium outputs. Other nutrients affecting output were energy and total fiber, which were associated with increased output. In another study [39], the effect of various foods on the amount of ileal excretion was measured in seven subjects who had normal functioning ileostomies. The patients were maintained on a self-selected control diet for three days and then the researcher assessed the effect on ileostomy function of 37 foods over the next three days. The foods tested were those often either interdicted for or avoided by ileostomates. The results, interestingly, showed that only seven of the foods (i.e., grapes, peaches, strawberries, bananas, raisins, prune juice, and baked beans) considerably increased the ileal effluent. Therefore, the above findings suggest that many foods and fluids may be needlessly restricted in this category of patients.

Some studies in the literature have observed that patients with a newly formed ileostomy often tend to avoid specific foods due to concerns of probable blockage or malfunction of their stoma [40-43] (Table 1 summarizes studies in the literature that concentrated on the effect of specific foods on ileostomy function). It has also been reported that people with ileostomies generally avoid high dietary fiber foods, fried foods, and food with skins, hulls, husks, and seeds. The reasons for avoiding these foods are fear of or previous experience with blockage, bloating, increased gas and odor, and constipation or diarrhea [42,33]. As far as alcohol is concerned, it has been suggested to increase loose, watery output [33]. It seems that more randomized controlled trials are required in order to shed more light on how different foods can affect ileostomy function. These patients are often encouraged to adhere to restrictions that are mostly based on health professionals’ experience and reporting patient feedback. Additionally, it is crucial to mention that individual tolerance may differ significantly; thus, patients should be advised to find out for themselves which foods provoke recurrent and consistent symptoms.

**Table 1:** Studies on the effect of specific foods on ileostomy function. FFQ, food frequency questionnaire; Na, sodium; MNA, mini nutritional assessment.

Study/author	Objective	Measured parameters	Findings/conclusions
de Oliveira et al., 2018 [40]	To evaluate the foods patients with a stoma avoid most frequently and the reasons for doing so.	Dietary intake was assessed using a quantitative FFQ. The FFQ was composed of 106 items that focused on 11 food groups.	Ileostomy patients avoided mainly vegetables and fruit due to concerns of increased appliance leakage and stoma output.
Kramer, 1987 [39]	To assess the effect of various foods on the amount of ileal excretion on patients with normal functioning ileostomies.	Subjects followed a certain test diet including foods that are often either interdicted for or avoided by ileostomates. The amount of ileal effluent was measured.	Only 7 of the 37 dietary substances tested significantly increased the ileal effluent.
McNeil et al., 1982 [38]	To determine factors that are likely to lead to metabolic disturbances in a group of ileostomy patients living at home.	Ileostomy output and composition, urinary and hematological indices of nutritional status, 7-day weighed record.	Ileostomy patients are at increased risk of salt depletion. Sugar intake was associated with reduced effluent and ileal Na outputs. Energy and total fiber were associated with increased output.
Migdanis et al., 2020 [41]	To assess the effect of a diverting ileostomy formation on the nutritional intake of patients with a newly formed ileostomy.	Nutritional intake was assessed using the 3-day dietary recall method.	The larger part of ileostomy patients (59%) reported avoidance of particular foods (legumes, wholemeal high fiber cereals, green vegetables, and fruits) due to fear of possible blockage or malfunction of their stoma.
Floruta et al., 2001 [42]	To collect data related to individual dietary recommendations and restrictions for people with ostomies.	A questionnaire was used that asked participants about food choices/avoidances that occurred because of their ostomy.	The majority of subjects (81%) reported that they did not have any ostomy management problems related to diet. The main foods avoided included fresh fruits, nuts, coconut, and vegetables due to increased output and gas.
Vasilopoulos et al., 2022 [43]	To assess the nutritional status of ileostomy patients pre- and post-operatively	MNA tool was used to assess nutritional status of participants.	Significant decrease in fruit and vegetable consumption postoperatively compared to preoperatively.

Anthropometry and body composition

It has been suggested that body composition is related to both post- and intra-operative complications in colorectal surgery as well as prolonged hospital stays. Studies have demonstrated that patients with a body mass index (BMI) of more than 25 kg/m^2^ have a higher risk of incisional hernias and an increased rate of surgical site infection [44,45]. It has also been reported that obese patients have an increased rate of developing stoma complications. In a retrospective analysis [46], when compared to patients of normal weight, obese patients experienced a higher overall number of stoma complications. The researchers reviewed 266 patients with 345 stomas (ileostomies and colostomies), and statistical analysis revealed that participants with a BMI of >30 kg/m^2^ presented a 47% complication rate compared to 36% among the BMI <30 kg/m^2^ patients (P < 0.05). In a more recent case-controlled study with a sample of 164 ostomates, multivariate analysis revealed that IBD and obesity were significantly associated with complications (odds ratio: 2.66; 95% confidence interval: 1.15-6.16). The most common complications experienced in obese patients were prolapse (21%), necrosis (21%), and skin irritation (21%), followed by infection (14%), stenosis (14%), and bleeding (7%) [47]. It is of great importance to state at this point that the majority of studies that assess the effect of obesity in colorectal surgery have not used standard criteria to define obesity. The ideal body weight equation (referred to as IBW) or BMI, mainly used so far, does have its limitations, as they are not reliable predictors of visceral adiposity and body composition. It has been suggested by several studies that BMI when compared with other methods of determining body composition, such as bioelectrical impedance, failed to classify obese men and women with a value of below 30 kg/m^2 ^[48-50].

The issue of whether indices of overweight predict body composition is important because body composition (i.e., total body fat percentage) rather than excess body mass is the more important health risk. In the case of stoma formation, technically obese patients have shortened, thick mesenteries, which preclude the ease of bringing the stoma to the skin through a thick subcutaneous layer. This can also increase the risk of retraction as well as necrosis, both of which can lead to pouching difficulties and skin irritation [51]. Thus, it seems as if visceral fat is a determining factor in stoma-related complications, and using a more accurate method of classifying adiposity, such as bioelectrical impedance or dual-energy X-ray absorptiometry (commonly known as DXA), would provide firmer conclusions on the impact of obesity in colorectal surgery and stoma complications. It should be mentioned that the obese patient represents a challenge for colorectal surgeons and in elective colorectal surgery preoperative weight loss should be recommended, when possible, in overweight patients in order to reduce the risk of complications associated with both the procedure itself and the formation of a stoma.

Concerning the effect of ileostomy on anthropometric characteristics of ileostomy patients, scientific data are very limited and contradictory. In a study with a cross-sectional design, no considerable differences were noted in body composition and anthropometric indices between patients with an ileostomy and a colostomy [40]. Additionally, in another cross-sectional study where patients with well-established ileostomies were compared with healthy matched subjects, no significant differences were observed in total body fat and body weight among the two groups [52]. In contrast, compared to normal controls, patients in a study by Pironi et al. [53] had a considerably higher frequency of anthropometric measurements below recommended values at the time their diverting ileostomy was closed. Moreover, in this study, a group of patients at the end of the defunctionalized stage had significantly lower mid-arm circumference (referred to as MAC), tricep skinfold thickness (referred to as TSF), and BMI values, in relation to a few months following their diverting ileostomy closure (7.6 months). Another pilot study assessing pre and post-operative nutritional status of ileostomy patients observed that weight, BMI, and arm/calf circumference noted significant decreases postoperatively compared to the preoperative stage [43]. Lastly, alongside Pironi et al. and Vasilopoulos et al., a late study also showed that a defunctioning ileostomy and hence, colonic exclusion seems to have an unfavorable impact on the body composition and anthropometric characteristics of this group of patients, during the first postoperative period compared to the preoperative stage [41]. More specifically, this study showed that mean total body fat percent, weight, and BMI significantly declined (from 28.6% to 25.6%, 75 kg to 71.6 kg, and 26.9 kg/m^2^ to 25.6 kg/m^2^, respectively) 40 days post-discharge from the hospital in an ileostomy group of 41 patients, as compared to their preoperative stage findings. Studies in the literature that concentrated on the effect of ileostomy formation on nutritional status markers are summarized in Table 2.

**Table 2:** Studies on the effect of ileostomy formation on nutritional status and intake. BMI, body mass index; SFM, skinfold measurements; BF, body fat; WT, weight; BN, body nitrogen; BP, body potassium; MAC, mid-arm circumference.

Study/author	Objective	Measured parameters	Findings/conclusions
de Oliveira et al., 2018 [40]	To compare the nutritional status of patients with an ileostomy or colostomy.	Anthropometric characteristics (BMI, SFM), body composition (BF %), energy and macro/micronutrient intake.	No differences between anthropometric characteristics, body composition and energy intake between the two groups. Ileostomy patients had significantly lower fat and niacin intake than colostomy patients.
Cooper et al., 1986 [52]	To compare the nutritional status between ileostomy patients with and without ileal resection and then compare both with healthy controls.	Anthropometric characteristics (WT, SFM), body composition (BF %, BN, BP), energy and macro/micronutrient intake, biochemical indices.	Ileostomy without ileal resection does not affect body weight and fat percentage, but leads to a reduction in total body nitrogen and potassium compared to healthy matched subjects.
Pironi et al., 1991 [53]	To assess the nutritional consequences of a defunctioning ileostomy and to compare them with those occurring in patients with a Brook-type permanent ileostomy.	Anthropometric characteristics (BMI, SFM, MAC), energy and macro/micronutrient intake, serum and urinary biochemical markers.	Patients at time of their ileostomy closure presented significantly lower anthropometric measurements than 7.6 months after the closure of the diverting ileostomy and compared to healthy controls. Authors recorded a significantly higher calorie intake in ileostomy patients throughout their defunctionalized stage compared to 7.6 months after the protective ileostomy closure, in the same individuals.
Migdanis et al., 2020 [41]	To compare the nutritional status between patients with a newly formed ileostomy and patients who underwent rectosigmoid resection without requiring a diverting ileostomy.	Anthropometric characteristics (WT, BMI), body composition (BF %), dietary intake, nutritional status biochemical markers.	Anthropometric/body composition characteristics and energy intake significantly declined in the ileostomy group 40 days following exit from the hospital compared to preoperatively.
Vasilopoulos et al., 2022 [43]	To assess the nutritional status and anthropometric characteristics of ileostomy patients pre- and post-operatively.	Anthropometric characteristics (WT, BMI, MAC), dietary intake, and nutritional status.	Anthropometric characteristics and dietary intake significantly decreased postoperatively compared to preoperatively.

Colon energy regulation and its effect on nutritional status

Despite the fact that the majority of nutrients are absorbed in the small intestine’s upper section, it has been indicated that the large bowel can also play a part in energy regulation and hence have an impact on the nutritional state of ileostomy patients. Several species of Gram-positive and Gram-negative bacteria exist across the digestive tract of humans, with a relatively small number of species being present in the stomach and small intestine [54,55]. In contrast, the colon contains a densely populated microbial ecosystem, with up to 10^12^ cells per gram of intestinal content [54]. Nutrients that were not absorbed by the small bowel, particularly dietary carbohydrates and, to a smaller extent, proteins and amino acids, are used as a nutrient substrate by the bacteria located in the large bowel for their growth. Short-chain fatty acids (i.e., propionic acid, acetic acid, and butyric acid) are produced as a result of the bacterial fermentation of proteins and carbohydrates, which can be used by several cells in the body and the bacteria themselves as energy substrates [56,57]. For instance, large intestines’ epithelial cells can absorb butyric acid and use it as an energy substrate. Colon can also absorb acetic and propionic acid, which are then transferred to other sites, providing energy sources for the brain, muscles, and liver cells [58-60].

The impact of an ileostomy on the energy balance and nutritional status of patients has also not yet received much attention in the medical community, probably due to the fact that, on most occasions, the small intestine remains intact. In an Italian study, the authors sought to assess the nutritional status of patients who had undergone an IPAA and had a protective ileostomy created [53]. The authors observed a significantly higher frequency of below-normal anthropometric characteristics of the patients with an ileostomy (at closure and three months after closure) compared to a group of healthy controls. The analysis of the data also revealed a significant improvement of all anthropometric parameters at 3 months and 7.6 months after the closure of the ileostomy, as compared to right after the closure time point. The authors attributed their findings to the non-occurrence of the absorption of short-chain fatty acids in the large bowel, due to colonic exclusion. The explanation behind this assumption was that after the closure of the protective ileostomy, the anthropometric measurements improved despite a decrease in the caloric intake, suggesting an enhancement of intestinal absorption.

Other factors that can affect energy balance or intake

Another important factor that has also been reported to affect the energy balance of ileostomy patients is the volume of ileostomy effluent. When the daily output from an ileostomy exceeds 1500-2000 mL/d (HOS-high output stoma), this is likely to have a significantly negative nutritional impact in this group of patients. When these losses occur long-term, ileostomy patients can suffer malnutrition, weight loss, and a negative energy balance [6,61]. The main causes for high output ileostomy include intra-abdominal sepsis, partial or intermittent bowel obstruction, enteritis (e.g., *Clostridium difficile *or *Salmonella *spp.), sudden drug withdrawal (e.g., steroids or opiates), administration of prokinetic drugs (e.g., metoclopramide), and recurrent disease in the remaining bowel (e.g., Crohn’s disease or irradiation bowel disease) [12,62]. Lastly, another parameter that needs to be taken into consideration is that some studies have shown that patients with a newly formed ileostomy often avoid specific foods, due to fear of potential blockage or malfunction of their stoma, thus leading to an impaired energy intake (Table 2). In a cross-sectional study where colostomy patients were compared to ileostomy patients, the researchers observed that significantly more people with ileostomy (20%) avoided foods such as vegetables and fruits compared to patients with a colostomy (4%), due to concerns about appliance leakage and increased stoma output [40]. In another study, the total energy intake of newly formed ileostomy patients significantly decreased postoperatively compared to the preoperative stage [42]. It was shown by the nutritional assessment performed, that a large proportion of patients with an ileostomy (59%) had the tendency to exclude various foods (mainly legumes, fruits, green vegetables, and whole meal high-fiber cereals) due to fear of potential stoma malfunction. Since the current literature on the impact of ileostomy (permanent or temporary colon exclusion) on energy and nutritional balance is generally limited, new studies are needed that will focus on the body composition and nutritional intake of such patients.

## Conclusions

Fluid and electrolyte abnormalities, resulting in secondary renal impairment, appear to be a common problem that requires further investigation in ileostomy patients. Future prospective randomized controlled trials examining measures that can prevent admission for rehydration (e.g., hydration methods or reduction of stoma output) are definitely required, as they can both improve post-operative life quality of the patients and reduce the monetary costs connected with rehospitalization.

In colorectal surgery diverting ileostomy formation may have an adverse impact on the dietary intake and general nutritional status of patients, particularly over the initial postoperative period. Dietary assessment and nutritional support when required might need to be incorporated in the typical clinical management of this patient group to avoid impaired energy intake and weight loss.

There are inconsistencies in the literature regarding foods that should be avoided by ileostomates. Many patients restrict their diets to exclude foods rich in dietary fiber (e.g., fresh fruits, vegetables, etc.) with their decision being supported and recommended by many clinicians. Lack of reliable information on the topic may lead these patients to become malnourished and to manifest a compromised nutritional status. Finally, more data and prospective studies are needed to facilitate the development of specific nutritional recommendations for this group of patients. Studies that would focus on assessing patients' nutritional intake (food diary, three-day recall) and investigate the effect of certain foods on the volume of ileostomy effluent and blockage or malfunction occurrence would enrich the existing body of literature regarding the relationship between diet and ileostomy function.
